# Growth and Nutrition of the Body

**Published:** 1875-02

**Authors:** Joshua B. Graves

**Affiliations:** Corning, N. Y.


Southern Medical Record.

Vol. V.—FEBRUARY, 1875.—No. 2.

### Original Communications.

### GROWTH AND NUTRITION OF THE BODY.

BY JOSHUA B. GRAVES, M.D., CORNING, N. Y.

*Read before the Meigs and Mason Academy of Medicine, and ordered to be published in the Southern Medical Record.*

Of late, there has been much written upon the subject of the growth and nutrition of the human body. Many new theories have been advanced, and things stated as *facts* which require *proof* before being thus received.

These new masters have taught that life is but a mode of ordinary force, and that the living thing differs from the non-living thing, not in quality, essence or kind, but merely in degree.

They do not attempt to explain the difference between a living thing and the same thing dead. They would (perhaps) tell us that *living* and *dead* are relative terms; that there is no *absolute* difference between the two states; and the thing which we call *dead* is, after all, only a few degrees less actively living than the thing we say is *alive.* This sort of reasoning is not very convincing, seeing that, although matter in the living state may suddenly pass into the dead state, this same matter can never pass back again into the living condition. Those who advocate this doctrine do not believe in the annihilation of force. When a living thing passes suddenly into the dead state, they do not tell us what becomes of the departing life-energy.

Professor Owen has lately avowed his belief in the doctrine that the so-called vital forces are really ordinary physical forces. He, however, admits that in one or two points proof is wanting. He maintains that the formation of living beings out of inanimate matter, by the conversion of physical and chemical into vital modes of force, is going on daily and hourly. The evidence he adduces to prove this strange theory is entirely unsatisfactory.

The different forms and properties of living beings can only be explained by supposing the influence of force different from ordinary forces, acting upon the matter of which they are composed, or upon the existence of properties transmitted or handed down from preexisting matter, having similar though not identical properties. These vital properties are superadded to matter temporarily, and are not, like the former, permanent endowments. The one class of properties remains permanently attached to the elements of matter. The other may be once removed, but can never be restored. The material properties belong to matter, dead or alive. But where are the vital properties in the dead material? If the above-mentioned theorists would restore the dead to life, we would soon become converts to their assertions.

In discussing the growth and nutrition of the human body, I shall accept the method of Dr. Beal, in his lecture at the Royal College in 1861, with regard to the division and classification of the component parts of the human body. He says:

"The body is made up thus: First, of living matter, called germinal matter."

This is the actual growing matter of all the tissues. It is not tissue, for it lives and grows. It may at length undergo a conversion into tissue. It is living matter, and by this term we mean that in this matter phenomena are observed which have not been explained, and cannot be accounted for by any known laws; which cannot be imitated artificially; which have never been observed except in living things. Among the peculiar powers of this living matter are—

1. The power of appropriating certain soluble matters, and of communicating to the properties which the living matter itself possesses.

2. The power of moving in all directions. One part of a living mass may move to another part. One portion may advance itself in front of another portion, or it may surround or encircle another portion.

3. This living matter has the power of causing the elements of matter to take up definite relations toward one another, so that definite compounds and definite structures may result, etc.

4. This living matter has the power of endless and definite increase. The balance of the human body is composed of formed material and pabulum.

The smallest portion of the body seen under a microscope consists of matter in the same state in each and every part. It consists, first, of living; second, matter formed from this, and pabulum which the living matter takes up. The matter in the first state is alone concerned in development and production of those materials which ultimately take the form of tissue, secretion or deposit, as the case may be. It alone has the power of growth, and of producing matter like itself out of material differing from it materially in composition, properties and powers. It is, therefore, called germinal or living matter. We thus distinguish it from formed material, which is in all cases destitute of these properties. The difference between these three component parts of the body is absolute.

The pabulum nourishes the body, but it is first converted into germinal matter. The ultimate particles of the pabulum pass into the germinal matter, and become alive like the matter into which they pass. The germinal matter forms the cells which are ultimately converted into formed material. There is now power in muscular fibre to appropriate the pabulum contained in the blood, and thereby to nourish itself. The germinal matter of all organisms, and of the tissues and organs of each organism, exhibits precisely the same character. It lives, and grows, and forms in the same way, yet the conditions under which the phenomena of life, growth and formation are carried on, differ very widely in different kinds of germinal matter. This is seen in temperature. The temperature in which one kind will grow and flourish, would be fatal to another kind. This is also true of the pabulum. The pabulum that will nourish one kind of germinal matter would destroy another. That the way in which the germinal matter moves, divides and subdivides, grows and undergoes conversion into tissue, is the same in all.

I wish, before proceeding in this discussion, to present to you a description of germinal matter, as seen under the microscope by Professor Beal—notwithstanding some gentlemen affect to despise the use of the microscope in the practice of medicine. "I see," says the Doctor, "under the microscope, a little, clear, transparent, structureless matter, which moves in various directions. Portions of the mass project at different points around the circumference. Some of these are again drawn into the general mass; others become detached, never to join again. Each separate mass grows, or takes up non-living matter around it, which non-living matter or certain of its elements becomes part and parcel of the growing, moving mass. The matter moves, and grows, and divides, and forms, and I find that everything that lives consists of matter like this, possessing like properties."

The Doctor further says: "I find no matter in nature which moves, grows, divides and forms, save matter that came from matter which did all these things before it, and therefore I call all matter which does all these things *living*, and matter which does *not* do these things *non-living*."

What we wish to know now is, why the matter grows, moves and forms? We are told that all this depends upon force, and that force is conditioned in the cell mechanism just as it is in a machine.

We answer, living matter came from living matter like itself, which lived before it, and this from pre-existing living matter. On the other hand, the machine was not derived from another machine after taking in material of which its elements are composed.

Force, of itself, cannot form the least possible machine or thing adapted to any definite end or purpose. What right, then, have men to say that force forms germinal matter, which, when formed, is more powerful than any machine ever formed by human effort or skill? In order to answer this question, it is not necessary for me further to pursue the subject of force, but will come back and take up the subject of formation of cells. It is generally supposed that when a tissue grows, certain matters existing in the blood pass from that fluid, undergo change, and are directly added to the tissue. In the nutrition of such a tissue as cartilage, it has been concluded that the intercellular substance is deposited directly from the blood, and that the masses of germinal matter or cells take no actual part in its formation. By what means, then, does the pabulum become altered, as it passes through the walls of vessels, and changed into cartilage? It is certain that there is not anything in the blood like cartilage already formed. The pabulum thus brought in contact with a tissue acts as any other foreign substance—it will produce irritation and inflammation. The pabulum of the blood has to be acted upon by some other power before it can nourish the tissues. That power, we assert, resides in the germinal matter. The living matter forms cells—these cells form the tissues of the body.

The pabulum of the blood is taken up by the germinal matter, and in its turn is gradually resolved into cells—these cells into tissues. Dr. Beal says "the existence of germinal matter before the production of formed material; the continuity of the germinal matter with the formed material in tissues in process of development; the circumstance of no case being known in which formed material is produced without germinal matter; and the demonstration that fluids will pass through a thick layer of formed material, and reach the germinal matter in the course of a few seconds—forced upon me the conviction that pabulum invariably passes to the germinal matter, and that it, or at least some of its constituents, undergoes conversion into this living substance, and acquires its properties and powers, while at the same time other portions of the germinal matter lose their active properties or powers, and undergo conversion into formed material."

Let us for a few moments consider the action of the cell. There can be no doubt that the action of many cells is due to the chemical changes excited by oxygen upon the formed material. This gas combines with some of the elements of a cell, and a new compound is formed, and is the secretion of the cell. Every one admits that oxygen is necessary to life. But how is it thus necessary to life? The principal demand for oxygen in living beings arises from the necessity for a chemical change, and the destruction of material which is formed in consequence of the vital changes occurring in the germinal matter. Oxygen acts principally upon the surface of the cells, upon the oldest formed material, rather than upon the germinal matter embedded in it.

The formed material is prevented from accumulating around the germinal matter of cells by external agencies. The most important of these agencies is oxygen. By the action of oxygen upon the formed material of the cell, it becomes more soluble, and is at once removed. This prepares the way for the pabulum of the blood to pass through the formed material to the germinal matter. It follows, then, that a cell may undergo the most active change without altering in size. The absorption of pabulum, and the production of new germinal matter, may be compensated for by the conversion of the latter into formed material, as the old formed material becomes oxydized and removed from the cell. Oxygen acts upon what we call the lifeless matter of the cell, and not upon the living germinal matter. Oxygen, then, does not support life directly, and yet is necessary to the continuation of life. It alone can convert the products of decay and death into soluble substances, which can be, and are, readily removed. This shows the fallacy of the doctrine that arterial blood is more nutritious than any other. The nutrient power of the blood does not depend upon the amount of oxygen it contains. You may ask, "Doctor, what does all this amount to?—what good will it do a practitioner of medicine?" Does it not teach us the nature of irritation and inflammation? If anything in the world can teach us the nature of disease, and in what way to remove the same, it must be a knowledge of the manner of the growth and nutrition of the body.

Now, suppose the hard formed material, which interferes with the access of pabulum to the germinal matter that composes the cell, is ruptured or softened, and the pabulum comes more readily in contact with germinal matter; what happens? The germinal matter increases. The cells multiply rapidly. The rupture or softening of the layer of formed material on the surface admits nutrient matter freely to the germinal matter, that, being set full, rapidly absorbs nutrient material. This is the change which results from irritation, and is essentially inflammation. How is pus formed? The microscope reveals this process. The mass of germinal matter, with the free access of pabulum to it, rapidly increases in size, and soon begins to divide into smaller portions. Parts move away from the general mass, and become detached, and thus form several separate masses of germinal matter. These masses become embedded in the formed material, instead of being proliferated into tissue or formed material. This action of the germinal matter produces a new material, and this is what we call pus. The pus corpuscle, then, is produced from the germinal matter of a normal cell which has been supplied with pabulum much more freely than in the normal state or situation. From what we can gather thus far, we may come to the conclusion that disease of the body may result in two ways. First, from the cells of an organ growing and multiplying faster than in the normal state. Second, from their growing more slowly than in the normal state. In the one case, the restrictions to growth are increased, and in the other they are decreased. Pneumonia or inflammation of the lungs may be adduced as a striking example of the first condition. In this disease the pabulum of the blood comes in contact with the minute atoms of germinal matter, and forms the exudation that we find in the air-cells of the lungs. All cases of atrophy of different organs are examples of the second. If the germinal matter is not supplied with nutriment, cells will not be formed and proliferated into tissue. There must, of necessity, be shrinking of the parts. These facts lead us to conclude that rate of the growth of cells in disease may be accelerated or retarded by changing the pabulum which is transmitted to them; also by changing the tissues through which it passes. Such considerations have a very important bearing upon the practical treatment of disease. Many of the so-called tonics have the property of coagulating albuminous fluid; also solutions of extracted matter, preparations of tannin and the neutral salts, the different acids and a host of remedies operate in the manner above described. Of all the remedies which operate to harden the tissues or to coagulate albuminous fluids, alcohol stands foremost. In what is called hemorrhagic diathesis, it is well known to be the most efficacious remedy we have.

This view of the subject shows us that bleeding and cathartics operate beneficially in certain diseases. They reduce the amount of pabulum in the blood, and are efficient in inflammation. This view of the subject gives us something that is tangible as a foundation for an opinion in medicine. If germinal matter is manufactured with too much rapidity, we can ascertain the fact and reduce the pabulum from which it is manufactured. If the disease is from the want of proper pabulum, we must increase the powers of digestion, and thereby furnish pabulum. Hence, you will perceive how it is that the ignorant quack and the scientific man will occasionally succeed in the practice of medicine. If the case be one in which there is a deficiency of pabulum, the quack nostrum that stimulates the digestive organs will be efficacious in relieving the patient. On the other hand, if there is too much pabulum, the quack with his "podophyllin, leptandrin," etc. (what they call rip gut), will reduce the pabulum in the blood, and thus cure the inflammation. The scientific physician will in one case bleed his patient, and thus remove very directly the pabulum from the blood. On the other hand, he will give tonics and stimulants. Therefore you perceive that the whole doctrine of alteratives is reduced to one single thing, and that is this: There are certain remedies, or I should say drugs, which operate upon the nervous system, and if you please, specifically upon particular nerves, and by that operation change the action of the system in conveying nutrition to the germinal matter, and in that sense they may be styled alteratives.

There is another point we must arrive at by this discussion, and it is that the phenomenon of growth and nutrition of the human body is from the hand of the Almighty, and is not the product of ordinary forces.

